# The New Medical Law—Another Interpretation

**Published:** 1880-10

**Authors:** 


### Editorial.

### THE NEW MEDICAL LAW.—ANOTHER INTERPRETATION.

In our last number we criticized somewhat severely the law enacted by the last legislature, regulating the practice of physic and surgery in this State, and pointed out the main features of each section. The criticism thus made was submitted to competent legal authority, who coincided in the conclusions at which we arrived. Some difference of opinion having arisen as to the question whether our interpretation of the law was correct, the opinion of the eminent jurist, Judge Clinton, was obtained by direction of the Buffalo Medical Association, which will be found in the published proceedings of a special meeting of that society, in this number. It will be seen that the venerable Judge differs from the views we advanced, concludes that "any person legally qualified to practice medicine and surgery in this

State, who does not register himself in compliance with the act, is prohibited from practicing, and made criminally liable by section three for practicing his profession." We gladly accept this view of the law, which makes it incumbent upon every physician to register, and also affords educated physicians and the public some protection against the quacks, now infesting almost every hamlet in our state. The question of the fruits of medical legislation was also referred to in our last issue, and some doubt expressed in regard to the results of this and previous enactments. We hope the present law in its operations will prove an exception to all previous efforts to afford protection to the medical profession. This Journal will co-operate with any effort made by responsible persons, or legally constituted societies, to make it productive, of good. It may be that the time has arrived for such an effort. The current of thought in professional circles has turned in the last decade towards a higher standard of medical education, and if among the fruits of this enlightened sentiment we can chronicle also favorable indications for the protection of the reciprocal interests of the profession and the public, we shall conclude that the times present an auspicious omen which in the near future will entitle to respect the diploma conferring upon its recipient the degree of Doctor of Medicine.

We trust that the opinion of the learned judge will be carefully perused by our readers, and the advice contained therein promptly acted upon. The medical profession has always manifested a deep respect for the laws of the State, and inasmuch as the present law is intended for its own protection, it should be cheerfully acquiesced in, and a movement inaugurated to execute its provisions, and thus accomplish the end sought in legislation upon this and kindred subjects.

